# Evidence of STLV-2 and STLV-3 infections in wild-living bonobos (P. paniscus ) from the Democratic Republic of Congo

**DOI:** 10.1186/1742-4690-8-S1-A92

**Published:** 2011-06-06

**Authors:** Steve Ahuka-Mundeke, Florian Liegéois, Octavie Lunguya, Valentin Mbenzo, Menard Mbende, Bila-Isia Inogwabini, Jean-Jacques Muyembe, Eric Delaporte, Martine Peeters

**Affiliations:** 1 UMI 233, Institut de Recherche pour le Developpement and University of Montpellier 1, Montpellier, France; 2 National Institute of Biomedical Research, Kinshasa, Democratic Republic of Congo; 3 Lac Tumba Project,WWF, Kinshasa, Democratic Republic of Congo

## Background

Among the four types of HTLV (1 to 4), only three have their simian counterparts (STLV-1, 2 and 3). STLV-1 and 3 have been found in a large number of captive and wild-living monkeys and great apes from Africa and Asia. STLV-2 was reported only in a limited number of captive bonobos (P. paniscus) from Democratic Republic of Congo (DRC) and despite its genetic distance with HTLV-2, STLV-2 is considered as a simian counterpart of HTLV-2. To date, no evidence of STLV-2 or any other known STLVs have been documented yet in wild-living bonobos. Given that bonobos are an endangered species, investigation of pathogens in this species in the wild is only possible by non-invasive sampling. Here we report a survey aimed at characterising simian retroviruses from wild-living bonobos in DRC.

## Methods

Between March and July 2010, fecal samples from wild-living bonobos (P.paniscus) were collected at Malebo forest in the Bandundu province located in the western part of DRC. Samples were collected in RNA-later. Species confirmation was done by mtDNA analyses. All faecal samples were screened for STLV infection using a generic PCR allowing amplification of partial fragment in tax (220bp). Sequence and phylogenetic analysis was done to confirm the STLV infection and identify STLV types. Attempts to amplfy other gene fragments were done to futher characterize the new STLV viruses.

## Results

A total of 268 fecal samples were collected in 2010 and mitochondrial DNA analyses confirmed that all the samples were from bonobos (P. paniscus). Overall, 3 (1.1%) out of 268 samples yielded positive amplification of tax fragment. Among them , one (Pp5538) was identified as STLV-2 and two (Pp5489 and Pp5560) as STLV-3 by phylogenetic analysis of tax fragment. Phylogenetic analyses of tax and LTR fragments showed that the new STLV-2 from Pp5538 clustered with STLV-2 strains previously described in captive bonobos.

All PCR attempts to amplify the LTR fragment of Pp5489 and 5560 samples were unsuccessfull.

## Conclusion

Our study shows that faecal samples can be used to screen for STLV infection in endangered apes, although most likely with lower sensitivities. We confirmed STLV-2 infection in wild-living bonobos and showed for the first time that bonobos are also infected with STLV-3 Additional work is warranted to fully characterize the new STLV-2 and 3 strainsa nd to document the STLV diversity on a larger number of wild bonobos from differnt geographic régions.

